# Evaluation and comparison of the effects of a new paste containing 8% L-Arginine and CaCO3 plus KNO3 on dentinal tubules occlusion and dental sensitivity: a randomized, triple blinded clinical trial study

**DOI:** 10.1186/s12903-024-04298-3

**Published:** 2024-04-29

**Authors:** Hamideh Sadat Mohammadipour, Hossein Bagheri, Saber Babazadeh, Mehrzad Khorshid, Zahra Shooshtari, Arsalan Shahri

**Affiliations:** 1 Department of Restorative and Cosmetic Dentistry, Mashhad Dental School, Mashhad University of Medical Sciences, Mashhad, Iran; 2 Dental Materials Research Center, School of Dentistry, Mashhad University of Medical Sciences, Mashhad, Iran; 3 Department of Community Oral Health, School of Dentistry, Mashhad University of Medical Sciences, Mashhad, Iran; 4 Independent Researcher, Mashhad, Iran; 5 Dentist, Research Assistant, Dental Research Center, Mashhad Dental School, Mashhad University of Medical Sciences, Mashhad, Iran; 6 Dental Materials Research Center, Mashhad Dental School, Mashhad University of Medical Sciences, Mashhad, Iran; 7 Mashhad dental school, Mashhad university of medical sciences university campus, P.O. Box: 9178613111, Mashhad, Iran

**Keywords:** Dentin permeability, Arginine, Calcium carbonate, Potassium nitrate, Tooth sensitivity

## Abstract

**Background:**

Dentin hypersensitivity, often occurring after dental treatments or from erosive lesions, is a prevalent patient complaint. This study introduces a paste combining 8% L-arginine, calcium carbonate, and potassium nitrate to evaluate its impact on dentinal tubules occlusion, dentin permeability, and tooth sensitivity.

**Methods:**

Dentin surfaces from 24 third molars (thickness: 2 mm) were divided into two groups of 12. One received the experimental paste, while the other received a placebo without desensitizer. Permeability and sealing ability were assessed through scanning electron microscopy (SEM) and dentin permeability measurement. The pastes’ effects on hypersensitivity were then examined in a triple-blind, randomized parallel-armed clinical trial with 16 eligible patients. Sensitivity to cold, touch, and spontaneous stimuli was recorded using the VAS scale at various intervals post-treatment. Statistical analysis was conducted using Shapiro-Wilk, Mann-Whitney U, Friedman, and Wilcoxon tests (α = 0.05).

**Results:**

The permeability test demonstrated a significant reduction in dentin permeability in the experimental group (*P* = 0.002) compared to the control (*P* = 0.178). SEM images revealed most dentinal tubules in the intervention samples to be occluded. Clinically, both groups showed a significant decrease in the three types of evaluated sensitivity throughout the study. However, no significant difference in sensitivities between the two groups was observed, with the exception of cold sensitivity at three months post-treatment (*P* = 0.054).

**Conclusion:**

The innovative desensitizing paste featuring 8% L-arginine, calcium carbonate, and potassium nitrate effectively occluded dentinal tubules and reduced dentin permeability. It mitigated immediate and prolonged dentin hypersensitivity to various stimuli, supporting its potential role in managing dentin hypersensitivity.

**Trial registration:**

http://irct.ir: IRCT20220829055822N1, September 9th, 2022.

## Background

Tooth sensitivity is one of the adults’ most common dental problems, characterized by short, sharp, and transient pain. In a meta-analysis study, the prevalence of tooth sensitivity ranged from 2.8 to 74% [1]. There is still no precise rationale for tooth sensitivity [2, 3]. Among all mechanisms suggested by now, Brannstrom proposed the theory of hydrodynamics (Fluid Movement/Hydrodynamic) in 1964. The aforementioned theory is the most acceptable theory based on the dynamic flow of dentinal fluid [4]. Usually, when dentin tubules are exposed to the external environment, different stimuli can make the fluid in dentin tubules to move, which leads to nerve fiber stimulation and pain [5]. The cause of tubule exposure is multi-factorial. Generally, the gradual wearing of enamel or gingival recession (which causes dentin or root surface cementum exposure, respectively) is the most common cause [6]. Usually, creating an outward flow caused by stimuli such as cold, dryness, and hyperosmotic solutions makes the tooth more sensitive than a flow toward the pulp (for example, by heat) [3]. The most common stimulus in dentin hypersensitivity (DH) is cold air flow, such as breathing in cold winter air or applying a dental unit air syringe [7].

Several active substances have been introduced for the treatment of DH. CPP-ACP (derived from cow’s milk protein, casein, calcium, and phosphate) [8], TCP (tricalcium phosphate, a calcium phosphate system which is stable in aqueous environments [9], Remin Pro (containing hydroxyapatite, fluoride, and xylitol) [10], Pro-Argin (containing arginine/calcium carbonate) [11], and NovaMin (containing calcium sodium phosphosilicate (CSPS)) [12] have shown positive results. Different commercial products have been offered for each of the aforementioned mechanisms, some of which contain one or more active ingredients that use only one anti-sensitive mechanism for treatment. Nevertheless, some of these substances treat sensitivity using two or more active agents, employing the effects of both mechanisms (physical blockage and nerve stimulation).

Arginine is an essential amino acid with an alkaline pH, and its dentin-desensitizing effect in combination with calcium carbonate (as a rich source of calcium ions) has been proven in previous studies, both *in-vitro* and in the clinic [11, 13–19]. Arginine and calcium ions, which are positively charged at physiological pH (alkaline by bicarbonate buffer), bind to the negatively charged dentin surface and create a calcium-rich layer [11].

By increasing the potassium concentration in nerve endings and preventing the generation of action potentials in interdental nerves, potassium salts have become the most popular desensitizer of dental nerves, whose dentin desensitizing effect has been proved in previous studies [20–24]. By causing depolarization, potassium salts prevent repolarization and transmission of pain messages through nerves. Potassium nitrate (5%), potassium chloride (3.75%), and potassium citrate (5.5%), all of which contain 2% potassium ions, are used as active ingredients [25]. Professional pastes, toothpastes, and gels containing potassium nitrate are specifically used to reduce the incidence of tooth sensitivity during the dental bleaching period. For this purpose, patient should start the treatment two weeks before the beginning of the bleaching period and continue treatment during the bleaching process.

To the best of the authors’ knowledge, no studies have evaluated the potential of the combination of Pro-Argin and KNO3 agents as a double-action anti-sensitive treatment. Few studies evaluating the combination of two mechanisms together have been published. Even those that utilized both mechanisms of action did not implement the Pro-Argin agent as an occluding mechanism [26].

Pain, as one of the permanent and apparent characteristics of dentin hypersensitivity, affects quality of life [27]. Recently, the Dentine Hypersensitivity Experience Questionnaire (DHEQ) was designed to evaluate the impact of DH on the quality of life. To prevent dentinal sensitivity recurrence, identifying etiological factors such as improper brushing technique, poor oral hygiene, premature occlusion contacts, gingival recession, and exogenous/endogenous acids is crucial [28]. However, due to the difficulty, impossibility and time-consuming nature of removing all the primary causes, the most common action in the clinic is the use of anti-sensitive agents in-office and recommend to keep using at home. This work aimed to evaluate the effect of a new combination paste containing 8% L-Arginine and CaCO3 plus KNO3 in treating DH in non-carious lesions. The null hypotheses tested in this study are as follows:

- There would be no difference between the two pastes with and without the desensitizing agents (8% L-Arginine and CaCO3 plus KNO3) in terms of dentinal tubule permeability.
- There would be no difference in reducing tooth sensitivity effectiveness between the two pastes.

## Methods

### New paste composition and production steps

The following steps were taken to synthesize a final volume of 20 ccs of 5% potassium nitrate paste and 8% arginine-calcium carbonate using the materials listed in Table 1. First, 1.6 g (gr) of arginine (Merck, Darmstadt, Germany) was added to a mixture containing 0.24 g of calcium carbonate (Dr. Mojallali Co., Iran) dissolved in 16 cc of distilled water (Kimia Co., Iran) and mixed to become homogeneous. The solution was mixed with a magnetic stirrer. Then 1 g of potassium nitrate (Merck, Darmstadt, Germany) and 4 cc of glycerin (Merck, Darmstadt, Germany) were added to the solution. Finally, to increase its wettability and reach the desired consistency, 0.3 g of CMC (Merck, Darmstadt, Germany) and 1.5 g of silica (Tetrachem Co., Iran) were slowly added to dissolve.

**Table 1:** The materials used in the present study to formulate the experimental anti-sensitive paste

Materials	Lot number	Role	Manufacturers
Arginine	K50676042	Anti-sensitive agent	Merck, Darmstadt, Germany
Calcium Carbonate	0127112112	Anti-sensitive agent	Dr. Mojallali Co., Iran
Potassium Nitrate	3,000,250	Anti-sensitive agent	Merck, Darmstadt, Germany
Distilled water	NA	Solvent	Kimia Co., Iran
Glycerol	K45347592	Humectant, Thickening agent	Merck, Darmstadt, Germany
Sodium Bicarbonate	K40235123	Buffer	Merck, Darmstadt, Germany
Carboxy Methyl Cellulose (CMC)	6,143,922	Thickening agent	Merck, Darmstadt, Germany
Silica	381,276	Thickening agent, Anti-sensitive agent	Tetra chem Co, Iran

NA, not applicable

**Fig. 1:**
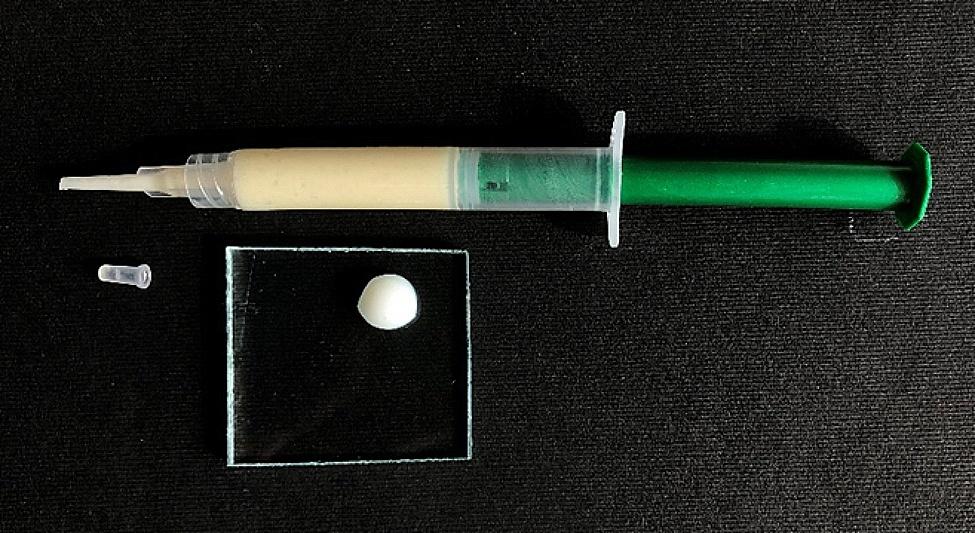
Formulated anti sensitive paste in applying syringe

The pH evaluation was accomplished; then, sodium bicarbonate (Merck, Darmstadt, Germany) solution was used to adjust the pH if needed. A digital pH meter (WTW Multiparameter benchtop meter inoLab® Multi 9620 IDS) was used. The final paste with 8–9 pH was packed in a tube and stored in a refrigerator for 24 h (Fig. 1).

### Study design

This triple-blinded, randomized, parallel-arm designed clinical trial with 1:1 allocation ratio, accompanied by a preclinical stage (*in-vitro*), was conducted at the Department of Cosmetic and Restorative Dentistry and Dental Materials Lab of Mashhad University of Medical Sciences, Iran, from June to August 2022. The study protocol was approved by the local ethical committee of the university (protocol number IR.MUMS.DENTISTRY.REC.1401.002) on April 6, 2022, in accordance with the World Medical Association Declaration of Helsinki. This study also followed the recommendations of the Consolidated Standards of Reporting Trials (CONSORT) statement [29]. The clinical trial was registered in the Iranian Registry of Clinical Trials (http://irct.ir) under the identification number IRCT20220829055822N1 on 09/09/2022. Before participation, the purpose of the study, risks, side effects, and benefits were thoroughly explained to the patients, and written informed consent was obtained from those willing to participate in the study.

### *In-vitro* phase (Assessment of dentine permeability)

**Fig. 2:**
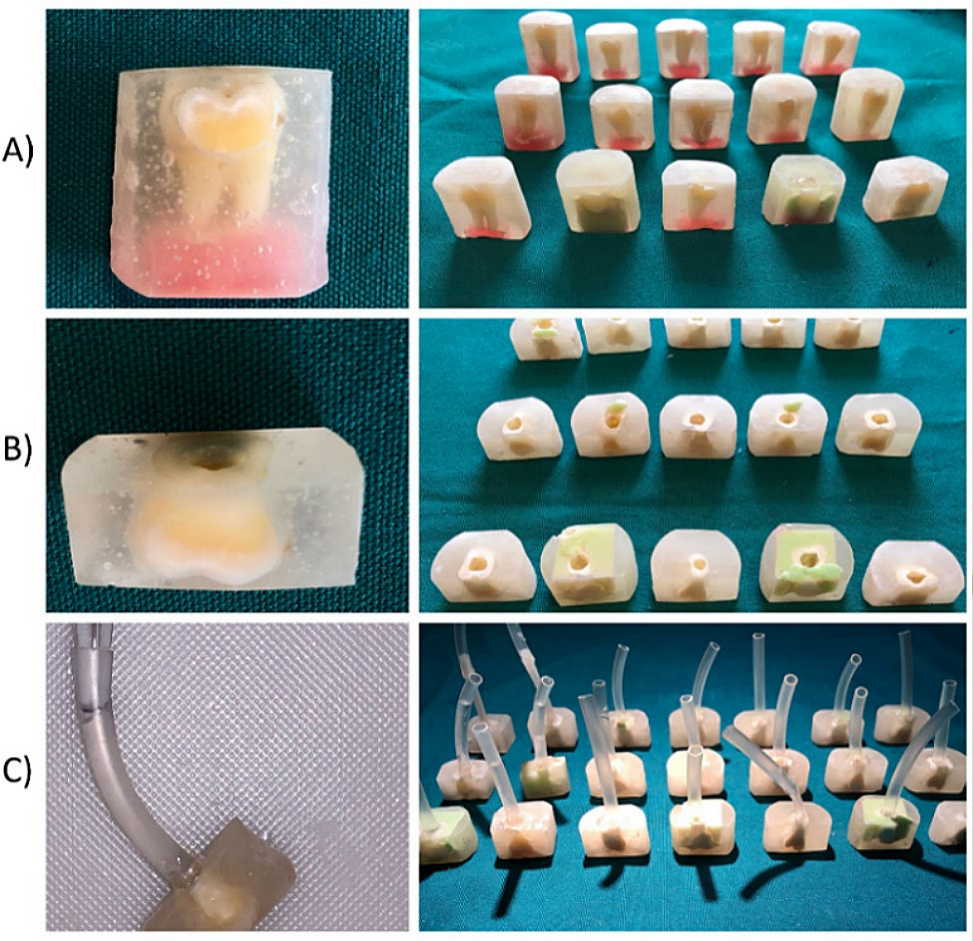
(**A**) Mounting the samples in self-cure acrylic resin and sandpaper used to reveal the dentinal part on the buccal surface. (**B**) Trimming the root section to access to the pulp chamber. (**C**) Cyanoacrylate glue was used to fix the laboratory tubing to the opening of the pulp chamber

Twenty-four human wisdom teeth with intact facial surfaces were selected for the study, with the sole exclusion criterion being the presence of any fractures. The teeth were disinfected in 0.5 wt% Chloramine-T for 72 h at 37 °C in an incubator, and any tissue remnants around the teeth were carefully removed. Subsequently, the teeth were vertically mounted (with the occlusal surface facing upward) in self-cured acrylic resin blocks (3 cm × 3 cm) from Marlic Co., Tehran, Iran. The buccal surface was meticulously trimmed using a trimmer (MESTRA RH-3000, Iran) until a smooth inner dentin surface was achieved. The exposed surface was further polished with sequential abrasive papers (800, 1000, and 1200-grit, Starcke; Germany) suspended in water to enhance the enamel’s smoothness (Fig. 2, (a)). All molars were carefully sectioned at the CEJ using the trimmer machine to provide access to the pulp chamber. After removing the pulp without touching the dentin walls, the pulp chamber was thoroughly rinsed with deionized water (Fig. 2, (b)). Subsequently, one side of the laboratory tubing was connected to the pulp chamber’s opening using cyanoacrylate glue, while the other side was connected to a pulp pressure simulator. If necessary, the opening at the bottom of the pulp chamber was widened using a turbine fissure bur (Diaswiss Co., Swiss) with water spray. To reveal the buccal dentin tubules, the remaining thin smear layer on the buccal dentin was etched by immersing it in a 0.5 M Ethylene Diamine Tetraacetic Acid (EDTA) solution (Merck Co., Inc, USA) for 2 min in an ultrasonic machine (Lyman Digital Ultrasonic Cleaner 2500 ml). Afterward, each sample was rinsed with deionized water for 30 s (Fig. 2, (c)).

**Fig. 3:**
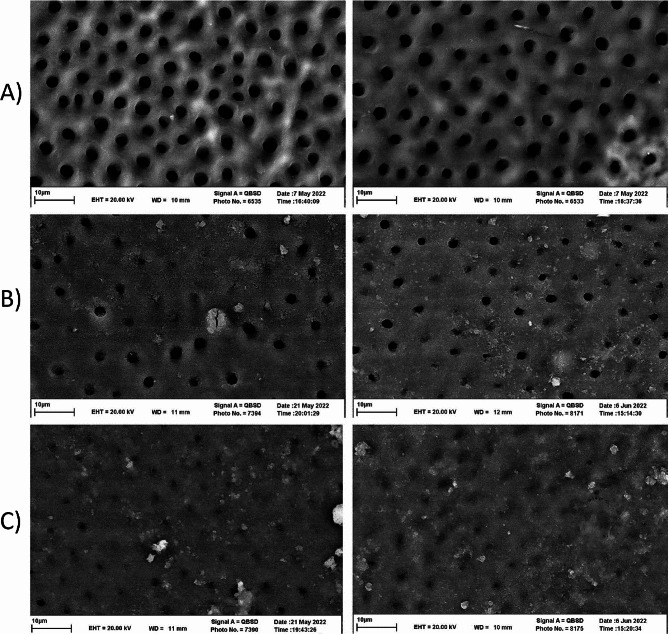
Microscopic images with a magnification of x5000. (**A**) Initial control sample to ensure tubules are opened before applying the paste. (**B**) Control paste. (**C**) Intervention paste

All the samples were numbered and divided into two groups (12 samples each) according to the proposed later treatment. At this stage, one sample from each group was used as a control for SEM, to ensure the smear layer’s removal and the tubules’ complete opening after immersion in the EDTA solution (Fig. 3, (A)). Other samples were kept in artificial saliva until the initial permeability measurement. The artificial saliva was formulated as previously described [30].

#### Group 1

New toothpaste containing Arginine and CaCO3.

#### Group 2

Base paste without any active agent (control group).

### Baseline data measurement

After group division, permeability measurements were performed. Primary permeability was assessed (Fluid Filtration Machine, Iran) for each sample with open dentin tubules using a water pressure of 70 cm (6.68 kPa). A ceiling suspended deionized water syringe supplied a hydrostatic pressure of 1 psi (70 cm H_2_O) through a micropipette to the pulpal chamber of the dentine samples via laboratory tubing. Before recording the permeability, the mixture was left for 2 min so that the liquid infiltrated into all dentin tubules. After 8 min, the amount of microleakage of water was read from the device to determine the primary permeability. The primary permeability of each tooth was compared with the permeability of the same tooth after the intervention. The baseline fluid flow (before the intervention) showed 100% permeability, and the permeability after the intervention was recorded as a percentage of the maximum amount.

### Application procedure

Twenty-four dentin samples prepared in the previous stage were randomly divided into two groups. In the intervention group, the formulated paste containing the active substance was used to reduce permeability, and in the control group, the base paste without the active substance was used.

The paste was used each day for 10 min for a week. At the same time while using the pastes on samples under the simulated pressure of the pulp, natural saliva was used on the surface of the buccal dentin to fully simulate the enzymes of the oral environment (including Arginine Deiminase). Natural saliva was prepared daily and before the application procedure. After 10 min of applying the paste, the dentin surfaces were washed for 30 s with running water, without pressure and any touch contact of surface, to remove the paste from the dentin surface. Each sample was transferred to a separate artificial saliva vial for storage until the next day in order to avoid dehydration. Artificial saliva was prepared based on the formulation of Zhou et al. [31], as previously described. The vials were kept in an incubator at 37 °C throughout the experiment. When each vial was removed from the incubator, it was allowed to equilibrate to ambient temperature for 30 min before measuring the liquid filtration.

### Final measurement

After 7 days, similar to the basic permeability measurement process, first we gave 2 min for the device to fill the tubules with water and equalize the pressure. Then, within 8 min, the microleakage was read to achieve final permeability.

### SEM phase (Assessment of tubule occlusion)

In addition to the two control samples that were previously scanned to ensure the opening of the tubules, two other samples from each group were randomly selected and prepared for observation by SEM. We employed SEM exclusively to enrich our findings qualitatively, thus, according to previous studies [32, 33], this sample size from each group sufficed. For this purpose, the samples were kept in 10% formalin solution for 48 h to stabilize. These samples were dehydrated in increasing concentrations of alcohol solutions containing 70, 80, 90, 95, and 100% alcohol, for 10 min each. After the dehydration process, the samples were first air-dried and then kept overnight in closed containers containing calcium sulfate (Drierite, W.A. Hammond, Xenia, OH, USA). The dried samples were mounted on microscope bases (SEM stubs). The mounted samples were observed using SEM (XL30, FEI, Hillsboro, OR, USA) at 10–20 kV, and photographs were taken of multiple areas at 5000x magnification. Finally, the open or closed tubules were checked under descriptive analysis (Fig. 3).

### Clinical phase (Assessment of dentine sensitivity in patients)

#### Sample size calculation

20 patients were included in the clinical part of this study, which was designed as a triple-blind clinical trial, according to the following criteria. According to the Split-Mouth method used in this study, each individual was both the intervention and control group simultaneously, makes each group size 20 and also reducing confounding factors.

#### Inclusion and exclusion criteria

Comprehensive dental examination was performed on all patients to determine their eligibility. The inclusion criteria are stated below:

- Patients with dentin hypersensitivity due to gingival recession and limited cervical lesions who do not need extensive treatment such as restoration.
- Patients aged 18 to 50 years.
- Patients in good general and oral health conditions.
- Patients not having a history of illness or long-term drug use.
- Patients not having a history of desensitizing treatment in the last six months.

#### Exclusion criteria

- The patients’ unwillingness to continue cooperation or who do not appear in visit sessions.
- Patients who use orthodontic appliances.

#### Randomization

Initially, ten men and ten women were selected to eliminate the effect of gender and the difference in pain tolerance. The randomization procedure was performed by www.random.org. An individual who was unaware of the research protocol created opaque consecutively numbered envelopes with information about the groups inside. The envelopes were opened by patients just before the treatment to select the right and left quadrants as the control or intervention group. We assessed the most sensitive tooth in each quadrant. Each participant’s measurements were done only in one jaw, either upper or the lower one.

#### Blinding

This study was designed as a triple-blind, in which the patient, evaluator, and statistician were blinded to the type of treatment. Both pastes were made with the same consistency, color, taste, and odor in identical syringes labeled A and B. A different researcher (not involved in the assessments) was responsible for the randomization process, applying the pastes based on codes A and B.

#### Study intervention

A retractor was used to retract the lips. In the control quadrant, paste without an anti-sensitive agent was used, and in the test quadrant, paste containing the anti-sensitive agent was used on the teeth. The anti-sensitive treatment process was performed in 2 visits on two consecutive days, each lasting for 30 min, to increase efficacy. While using the paste in the mouth, suction was used to control saliva flow, but the teeth were not thoroughly dried, so that some small amount of saliva could provide natural enzymes. Since the purpose of producing this material was to be easily used by patients at home, and not to require any specialized equipment, the paste was applied by finger and rubbed for 60 s and three times during the 30 min treatment session.

In order to standardize the oral hygiene conditions of the patients during the study, each was given a daily toothpaste containing 1450 ppm of fluoride (Pooneh, Iran) to use at least twice a day. This way, the disruptive effect of other dental desensitizers during the study was minimized. Patients were also asked not to use mineralizing substances, mouthwashes, or other toothpastes duringthe treatment period.

#### Sensitivity evaluation

The Visual Analogue Scale (VAS) was used to evaluate dental sensitivity. Before the start of the treatment (T_0_) and immediately after the end of the treatment on the second day (T_1_), the level of sensitivity to stimulating stimuli and spontaneous sensitivity to different stimuli were recorded. The assessment of sensitivity to cold stimulus was done by directing a 3-second application of compressed air from a triple air dental syringe perpendicular to the cervical third of teeth from an approximate 1-cm distance. During the assessment of cold sensitivity, adjacent teeth were covered with cotton rolls so that they would not be affected. Central teeth were not evaluated due to the possibility of interfering with the perception of sensitivity by the patient. However, non-evaluated teeth also received anti-sensitive treatment to take ethical considerations into account.

24 h (T_2_), one week (T_3_), one month (T_4_), and three months (T_5_) after the treatment, the patients were asked to visit return, and the level of dental sensitivity was reevaluated. At the end of the evaluations, if requested by the patient, treatment was also performed for other sensitive teeth.

### Statistical analysis

The in-vitro data was normally distributed while the clinical data were not, as determined by the Kolmogorov Smirnov test. Statistical analyses were conducted using SPSS version 26.0 software, with a 5% significance level. For Phase 1 and 2, dentin permeability was measured as a percentage of base permeability, considered 100%. Repeated measures ANOVA and the paired T-test were used to compare study groups and fluid infiltration results, respectively. SEM images were qualitatively compared. Sample size was set to 12 per group, based on Zhou et al.’s study and assuming 80% test power and a 5% type I error rate. For Phase 3, due to the non-normal distribution of data, sensitivity was classified as painless (score 0), weak (scores 1–3), moderate (scores 4–6), and high sensitivity (scores 7–10). Non-parametric tests were used to compare these categories. Changes over time and from baseline were assessed using the Mann-Whitney, Friedman, and Wilcoxon Signed Ranks Tests, respectively. The data were visualized with tables and graphs. Twenty participants were selected for the clinical study, allowing for dropouts while ensuring statistical power.

## Results

### Phases 1 and 2

The primary and final dentin permeability were calculated, and by dividing these numbers, the final permeability was obtained in microliters per minute. The before and after permeability results in each group were compared with Paired T-test and final permeabilities are reported as a percentage of initial permeability in Table 2. As shown, the decrease in permeability was significant only in the intervention group (*p* = 0.002) and not in the control group (*p* = 0.178).

**Table 2:** Comparison of permeability reduction in intervention and control groups

Group	Final permeability (%)	Mean difference ± SD	*P* value (paired t-test)
Intervention	58%	0.86 ± 0.70	0.002*
Control	75%	0.59 ± 1.35	0.178

*: Significant at *P* ≤ 0.05

As shown in Fig. 3, in part A, the openings of the dentin tubules are completely open and the smear layer and smear plugs have been removed. In part B, the base paste without anti-sensitive active ingredient is used, it covers the surface and closes the opening of some dentin tubules, but open dentin tubules are still visible. In part C, where the paste with the active ingredient Arginine/Calcium Carbonate along with Potassium Nitrate is used, the surface coating is thicker and more uniform. Furthermore, it almost closed all the opening of the dentinal tubules in many areas. It indicates the sealing properties of the intervention paste.

### Phase 3

#### Participant characteristics

**Fig. 4:**
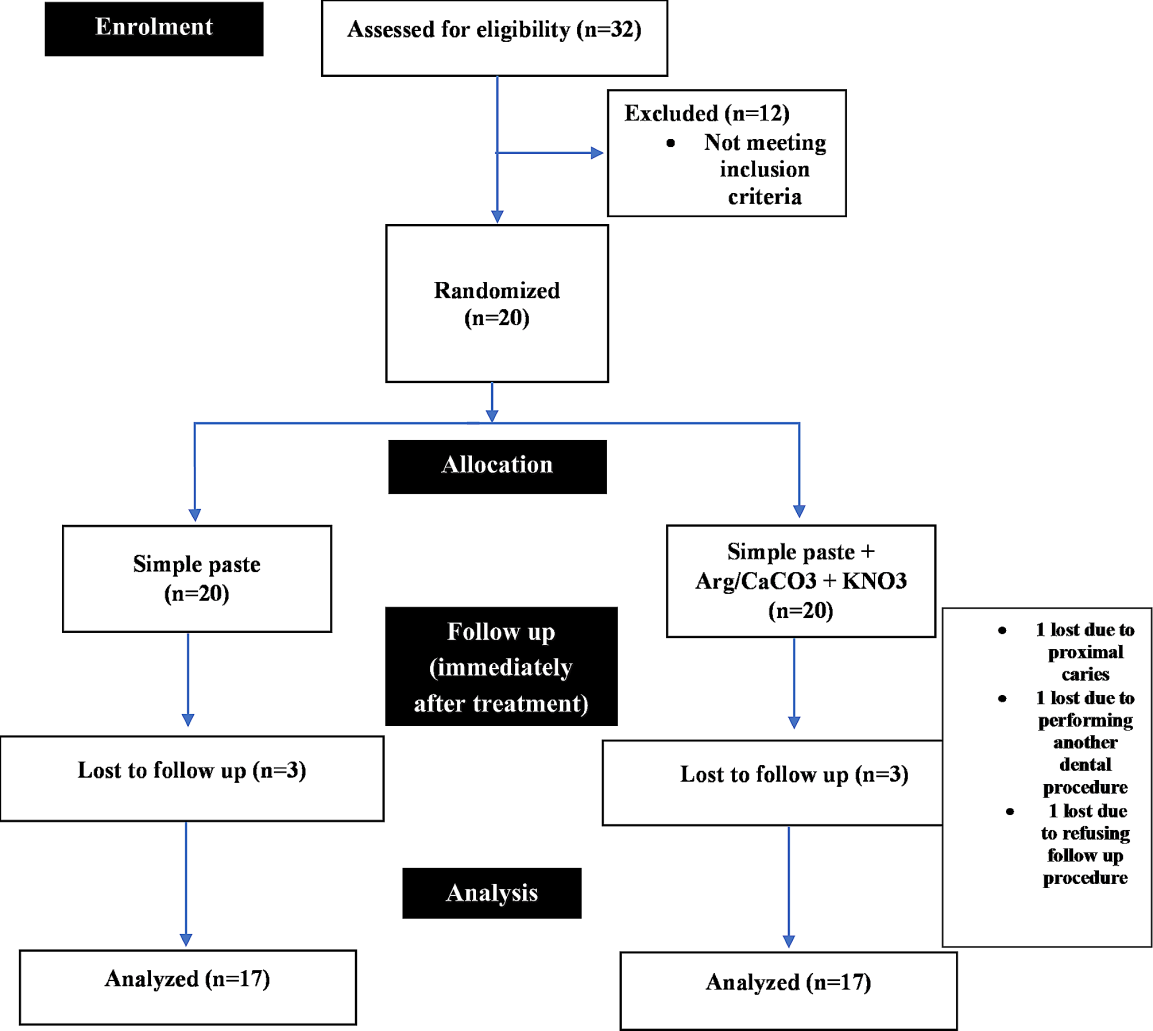
Flowchart diagram showing enrollment, allocation, follow-up, and analysis during the study, based on the CONSORT statement

The clinical trial started with 20 patients, including ten men and ten women. The participants’ mean age was 25.75 ± 4.25 years (range between 21 and 44 years). Unfortunately, three patients have not participated in the final evaluation. They were excluded (one due to a diagnosis of proximal caries, one due to performing another dental procedure during the follow-ups, and one due to not cooperating with the follow-ups). Therefore, data from 17 subjects were gathered and analyzed after three months (Fig. 4).

#### Within-treatment sensitivity comparisons through time

To compare the sensitivity values, nonparametric analyses were used due to the lack of normality in the data (*P* < 0.050).

**Fig. 5:**
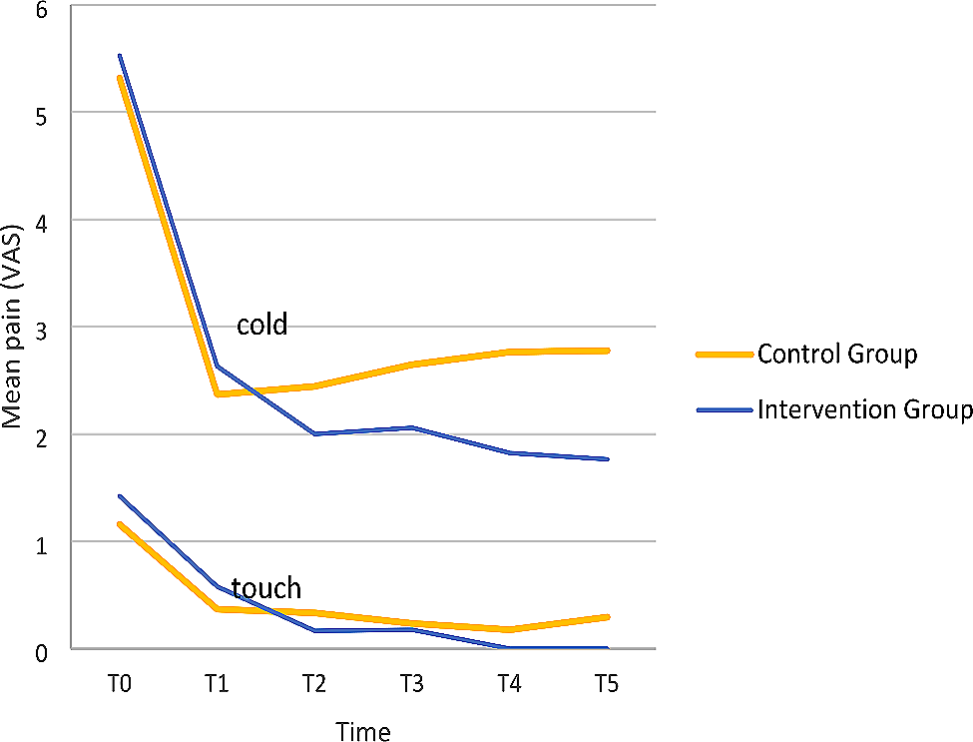
Sensitivity to cold and touch through time

The Friedman analysis of the VAS score results indicated that the intervention paste significantly decreased cold and tactile sensitivity immediately after the treatment (*P* = 0.001), while the control paste significantly decreased the cold sensitivity only. The Wilcoxon rank test indicated that from T1 onwards, the difference between follow-ups was insignificant in either group. Also, no significant difference was observed in terms of spontaneous sensitivity in either group (Table 3). The data are visualized clearly in Fig. 5.

**Table 3:** Within-treatment sensitivity comparisons through time

Group	Time Point	Cold sensitivity		Tactile sensitivity		Spontaneous sensitivity	
		Mean Rank	Friedman Test	Mean Rank	Friedman Test	Mean Rank	Friedman Test
Control	T_0_	5.21 ^a^*	χ2 = 32.40 *P* < 0.001	4.09	χ2 = 9.47 *p* = 0.092	4.09	χ2 = 5.000 *p* = 0.416
	T_1_	2.91 ^b^		3.59		3.59	
	T_2_	3.21 ^b^		3.47		3.47	
	T_3_	3.21 ^b^		3.29		3.29	
	T_4_	3.29 ^b^		3.26		3.26	
	T_5_	3.18 ^b^		3.29		3.29	
Intervention	T_0_	5.53 ^a^	χ2 = 47.41 *P* < 0.001	4.24 ^a^	χ2 = 20.60 *P* < 0.001	3.74	χ2 = 7.14 *p* = 0.210
	T_1_	3.47 ^b^		3.71 ^b^		3.56	
	T_2_	3.12 ^b^		3.41 ^b^		3.56	
	T_3_	3.15 ^b^		3.41 ^b^		3.38	
	T_4_	2.94 ^b^		3.12 ^b^		3.38	
	T_5_	2.79 ^b^		3.12 ^b^		3.38	

*: Different lowercase letters indicate significant differences within each group (according to the Wilcoxon Signed Rank Test)

#### Sensitivity comparison among groups at different time points

When comparing groups using Mann-Whitney analysis, no significant difference was observed. But a different long-term trend was observed regarding only cold sensitivity. To be more specific, in terms of cold sensitivity, the intervention group experienced a decreasing trend till the last follow-up. But recurrent sensitivity was observed in the control group in long-term (Table 4).

**Table 4:** Cold sensitivity comparison among groups at different time points

Time	Group	No sensitivity *N* (%) *	Mild sensitivity	Moderate Sensitivity	Severe sensitivity	Total	Mann-Whitney Test
T_0_	Control	0 (0)	2 (10.5)	14 (73.7)	3 (15.8)	**(100)19**	*Z = 0.527* *P* = 0.598
	Intervention	0 (0)	5 (26.3)	10 (52.6)	4 (21.1)	**(100)19**	
T_1_	Control	5 (26.3)	8 (42.1)	6 (31.6)	0 (0)	**(100)19**	*Z = 0.411* *P* = 0.681
	Intervention	3 (15.8)	10 (52.6)	6 (31.6)	0 (0)	**(100)19**	
T_2_	Control	5 (27.8)	6 (33.3)	7 (38.9)	0 (0)	**(100)18**	*Z = 0.438* *P* = 0.662
	Intervention	5 (27.8)	8 (44.4)	5 (27.8)	0 (0)	**(100)18**	
T_3_	Control	4 (23.5)	6 (35.3)	7 (41.2)	0 (0)	**(100)17**	*Z = 0.660* *P* = 0.509
	Intervention	5 (29.4)	7 (41.2)	5 (29.4)	0 (0)	**(100)17**	
T_4_	Control	2 (11.8)	9 (52.9)	6 (35.3)	0 (0)	**(100)17**	*Z = 1.183* *P* = 0.237
	Intervention	5 (29.4)	8 (47.1)	4 (23.5)	0 (0)	**(100)17**	
T_5_	Control	3 (17.6)	8 (47.1)	6 (35.3)	0 (0)	**(100)17**	*Z = 1.925* *P* = 0.054
	Intervention	6 (35.3)	7 (41.2)	4 (23.5)	0 (0)	**(100)17**	

*Number and percentage of subjects

In terms of changes in cold sensitivity over time, unlike the control group, where sensitivity recurred over time, the trend of sensitivity in the intervention group was downward until the third month, and a decrease in sensitivity was maintained in patients.

## Discussion

The *in-vitro* and clinical investigation of the anti-sensitivity paste effectiveness containing L-arginine/calcium carbonate 8% and potassium nitrate demonstrated that the paste decreases the permeability, closes the dentinal tubules, and significantly reduces dentinal hypersensitivity (DH) regarding cold and tactile sensitivity in long term (3 months after treatment); therefore, the first null hypothesis was rejected. DH is linked to dentinal tubule permeability, resulting from factors like occlusal wear, deep caries, brushing abrasion, erosion, and parafunctional habits. Commonly, anti-sensitive toothpastes are used to alleviate DH and reduce patient discomfort [34].

According to the *in-vitro* results of this study, the initial average permeability of the study groups did not display significant differences. However, a noteworthy reduction in dentin permeability was observed in the intervention group after one week of paste use, indicating the effectiveness of the formulated paste in closing dentinal tubules. Consequently, the second null hypothesis of the research is rejected. These findings were further supported by microscopic examination (at X5000 magnification), which revealed predominantly open dentinal tubules in the control group, whereas the intervention group exhibited almost complete closure of the tubules, thereby reinforcing the study’s results.

This finding aligns with Parmar et al.’s study [18]. Their electron microscope analysis showed more closed tubules in the arginine group than in the control. Midha et al. [35] found positive effects for all anti-sensitive agents, with NovaMin, followed by arginine and potassium nitrate, most effective in closing dentine tubules. In contrast to Midha et al., Rajguru et al. [36] reported arginine paste as more efficient in closing tubules but found NovaMin to resist acid challenges better. Since the decrease in the permeability of dentinal tubules in laboratory conditions may be different from the changes that occur in the biological, complex, and dynamic environment of the mouth; further investigations under clinical conditions are necessary, that’s why this project was continued with a clinical investigation (phase 2).

Hydraulic conductivity refers to liquid transfer across a surface unit under pressure over time. Lab studies identify factors influencing dentin permeability, with various methods proposed for measurement [37–40]. Dentin disc-based tubule closure assessment is the gold standard for hypersensitivity [41, 42]. In this study, SEM analysis followed dentine permeability assessment, and clinical evaluation included touch, cold, and spontaneous sensitivity.

According to the hydrodynamic theory, there are two primary approaches to treating DH: (1) closing dentinal tubules to minimize fluid flow in response to stimulation, and (2) reducing excitability in interdental nerves to minimize their response to fluid movements [43, 44]. Various commercial products are available for each mechanism, containing either single or multiple active substances, targeting only one anti-sensitive mechanism or employing a combination of methods (physical blockage and nerve stimulation).

In this study, the intervention group used a paste containing 8% L-arginine/calcium carbonate and potassium nitrate, while the control group used a base paste without the active anti-sensitive substance. Arginine, with an alkaline pH, has been proven effective in combination with calcium carbonate [13, 14]. It binds strongly to negatively charged dentin surfaces due to its cationic nature, forming rapid hydrogen bonds and create a calcium-rich layer [13, 14, 45]. The arginine-calcium carbonate method involves natural processes that block dentinal tubules with calcium-rich materials [11]. In contrast, the potassium nitrate approach elevates potassium concentration in nerve terminals, preventing action potentials in interdental nerves and pain message transmission by causing depolarization and inhibiting repolarization. Potassium nitrate (5%), potassium chloride (3.75%), and potassium citrate (5.5%), all of which contain 2% potassium ions, are used as active ingredients [25].

According to the clinical results of the present study, the study found that both groups experienced immediate cold sensitivity reduction after treatment, more pronounced in the intervention group, which also showed sustained long-term anti-sensitive effects compared to the base paste, over time. Unlike the control group, where sensitivity recurred over time, the sensitivity trend in the intervention group was downward steadily, maintaining lower levels until the third month. In terms of touch sensitivity, the intervention group consistently showed significant reductions over time, maintaining lower levels than before treatment. The same figure in the control group witnessed insignificant changes over-time, except immediately after the treatment. The two control and intervention groups showed no significant difference at each time point. For examining spontaneous sensitivity, there was no significant difference between the two groups at any time. In the intra-group comparison over time, as well as in the two-by-two before-after comparison, the level of spontaneous sensitivity did not show any significant changes.

Considering the noteworthy reduction in cold and touch sensitivity observed in the intervention group, this study aligns with previous research demonstrating the effectiveness of Pro-Argin-based pastes, both immediately after treatment and in subsequent evaluations at 3 days [46] or 2 weeks later [19, 47]. Hall et al. [48] examined the long-term effects of NovaMin and Pro-Argin pastes over 11 weeks, revealing significant sustained sensitivity reduction in agreement with the present findings. Investigations into the impact of potassium on sensitivity reduction, both individually [24] and in combinatio with fluoride ions [22], consistently corroborate each other and the current study, confirming the positive influence of potassium ions. In a study, Tolentino et al. [23] explored the effects of potassium nitrate and low-power laser, separately and concurrently, on dentin sensitivity reduction. They found that the combined treatment of these mechanisms proved more effective than individual approaches, suggesting a multi-session protocol with a minimum of three consecutive sessions to maintain prolonged desensitization. Mahesuti et al. [49] compared UltraEZ, a 5% potassium nitrate-based paste, to MI Paste containing CPP-ACP. They concluded that while potassium nitrate offered short-term and rapid effects, MI Paste provided slower but longer-lasting effects through tubular blocking. Although few studies have addressed spontaneous sensitivity, likely due to its measurement method’s unreliability in evaluation sessions, there remains an unexplored avenue for investigating the combined effect of arginine and potassium, despite the established efficacy of each substance independently.

The Air Blast test measured cold sensitivity, and probe movement assessed touch sensitivity. The Air Blast test is a common, controllable and repeatable method for this purpose [50]. The Visual Analog Scale (VAS) was utilized to recorded dental sensitivity at various times. VAS is an accurate scale for tooth sensitivity diagnosis based on previous studies [51, 52]. An issue with VAS is its reliance on individual pain tolerance. To solve this, the Split-Mouth method was employed, making each individual both the intervention and control group simultaneously, which consequently reduces the negative impact of confounding factors. Several studies have previously used and proven the reliability of the split-mouth method to assess tooth sensitivity [53, 54].

A clinical trial should have an appropriate duration to observe the maximum effect of the active substance while minimizing placebo effects and confounding factors. In this study, we measured sensitivities at six time points: before treatment (T0), immediately after treatment (T1), 24 h (T2), one week (T3), one month (T4), and three months after treatment (T5). This three-month follow-up aligns with recent studies, such as Bae et al.’s review [50], where the long-term effects of anti-sensitive paste were evaluated over periods of four to twelve weeks.

Based on Wilcoxon test results showing significant sensitivity improvement over time, it’s evident that a single use of anti-sensitive substances reduces patient discomfort, and continued home use maintains treatment benefits. While previous studies recommend three days’ home use after in-office treatment [55], some studies extend this period to two weeks for optimal results [19, 22, 56, 57]. However, this extended home use presents challenges due to uncontrollable factors and variations in brushing methods. In this study, with participant consent for additional visits, in-office treatment was conducted in two 30-minute sessions on consecutive days.

In the current study, as many previous studies [19, 24, 56, 58], the reduction in sensitivity in the control group was also significant, with the intervention group showing a more significant and sustained decrease over time. Reduced sensitivity in control groups is common, influenced by factors like placebo effects [59, 60], participant awareness of the study [61], and the Hawthorne effect [56], where improved oral hygiene during the study period may contribute. The base paste’s components, such as silica, may contribute to dentin surface layer formation and permeability reduction [22]. Despite these effects, the base paste formulation retained silica for its benefits, including thickening and sensitivity reduction, aligning with intended market release.

In previous literature, physical blockage of tubules has proven more effective than reducing nerve excitability [35, 56, 62]. Studies show mixed results for desensitizers with potassium ions, with some expressing doubt [63] and others confirming their effectiveness [49, 64–69]. No clear superiority has been established between these methods, suggesting that combining both approaches may yield better outcomes. Although dentinal tubule closure is a common approach in anti-sensitive toothpaste, no product permanently achieves this. Potassium ions also require prolonged use (2 weeks, up to 8 weeks for significant relief) [70], and there is debate about their effectiveness because they have to move inward to reach the pulpal nerves against the direction of the flow of dentin fluid to reach their point of action [71]. Thus, neither method alone is ideal.

The ideal goal for reducing sensitivity involve immediate pain relief while maintaining long-term effects. Most studies focus on the short-term impact of substances using the physical blockade desensitization method. For instance, clinical trials demonstrated that an 8% arginine and calcium carbonate combination (Pro-Argin Technology) provided “immediate” relief after just one application [72]. Another study by Minkoff et al. [73] examined strontium chloride’s desensitization effects, which were noticeable immediately and lasted for two more weeks. Conversely, the potassium ion mechanism often targets long-term relief. Bartold et al.’s study on potassium nitrate noted its cumulative effects taking weeks for a significant reduction in sensitivity [74]. The paste in this study achieved both goals, reducing short-term sensitivity (immediately and 24 h post-treatment) and maintaining effects in the long term (the downward trend until the final three-month evaluation), aligning with prior research on this topic.

This study combined two desensitizing methods using two substances to make it more effective. Although, chemically, no adverse reaction occurs between these two substances, but the immediate sealing of arginine/calcium carbonate may disturb the penetration of potassium ions and this possible interference must be considered. Limited research has simultaneously combined desensitizers to address dentin sensitivity. For example, Sowinski et al. [22] used a two-chamber syringe to administer potassium nitrate with stannous fluoride, ensuring separate paths with different pHs mixed only during application to avoid chemical interference. Parmar et al. [18] showed that initial sensitivity reduction with Pro-Argin paste is related to fully closed tubules, while semi-open or open tubules gradually close with repeated paste use, facilitating potassium ion penetration to reduce sensitivity. Our electron microscope images, aligned with prior research, revealed not all tubules closed with the Pro-Argin mechanism, leaving some open for potassium ion penetration. Sowinski et al. [22] indicated that these methods not only avoid interference but also enhance potassium’s effect, creating a layer that reduces fluid stimulation in tubules and allowing faster potassium ion travel to nerve terminals, especially in response to stimuli like cold, drying, and hyperosmotic solutions, causing tubular fluid outflow [75]. Another recent study also assessed the efficacy of a biomimetic nano-hydroxyapatite remineralizing solution on a hypomineralized enamel surface and its effect on enamel microhardness. The application of nano-hydroxyapatite solution induced a significant *in-vitro* decline of demineralized areas after the first week of application. Conversely, no significant differences were seen between untreated enamel surfaces and remineralized surfaces after 2 months of remineralizing treatment. Remineralized enamel showed significantly higher microhardness figures than demineralized enamel, but lower figures than intact enamel [76].

### Limitations and future suggestions

The study had limitations due to a small number of patients and a limited treatment schedule. Economic and time constraints hindered conducting multi-session treatments, and the Fluid Filtration device lacked full standardization for research purposes, affecting the results. To standardize in-vitro specimens after abrasive papers use on the buccal height of contour, we had to assess the mid-buccal dentine. On the other hand, cervical dentine was assessed in clinical phase because most of the samples had tooth sensitivity due to gingival recession.

Future studies should investigate the efficacy of low-power laser therapy as a complementary approach to manage dentinal hypersensitivity, especially in cases of severe sensitivity. Additionally, thorough evaluations of sensitivity-reducing agents are advised to ensure their effectiveness in resisting acidic conditions. To enhance the credibility of sensitivity research, innovative study designs should be adopted to minimize the impact of the placebo effect.

## Conclusion

The experimental paste containing 8% L-Arginine and CaCO3 plus KNO3 successfully decreased the dentinal permeability in-vitro. In addition, in the clinical phase of the study, the paste was successful at reducing tooth sensitivity either immediately (short-term) or in long-term. This shows its potential as a proactive action to prevent or treat tooth sensitivity. As such, it can be recommended for using in-office by the operator or at-home by patients in order to maintain and enhance the beneficial effects.

## Data Availability

The data of the current study are available from the corresponding author on reasonable request.
